# Experimental models for developing oncolytic virotherapy for metastatic prostate cancer

**DOI:** 10.3389/fimmu.2025.1626432

**Published:** 2025-07-10

**Authors:** Ying-Cheng Chen, Marxa Leão Figueiredo

**Affiliations:** Department of Basic Medical Sciences, College of Veterinary Medicine, Purdue University, West Lafayette, IN, United States

**Keywords:** oncolytic virus, metastatic, prostate cancer, immunotherapy, virotherapy, experimental, models

## Abstract

Cancer has remained the second leading cause of death worldwide for over a century. Despite significant advances, effectively targeting cancer cells and overcoming therapeutic challenges remain critical goals. In this review, we focus on advanced metastatic prostate tumors, where the patients’ five-year survival rate is less than 35%. While standard androgen deprivation therapy (ADT) has been effective for most prostate cancer patients, recurrence of aggressive tumors is common, emphasizing an urgent need for new treatment strategies. Immunotherapy has gained attention for its potential to harness the immune system against cancer cells. Among these, oncolytic virotherapy stands out for its tumor-specific tropism, its ability to transform or convert the immune-suppressive tumor microenvironment by enhancing immune cell infiltration, and its capacity for therapeutic gene delivery. This review explores the background of commonly used viruses, evaluation models (including cell culture, animal models, *ex vivo* platforms, and clinical trials), and the anticipated outcomes and challenges of oncolytic virotherapy. By addressing these aspects, we aim to provide a comprehensive overview of the current state and future directions of oncolytic virotherapy models in the treatment of advanced prostate cancer.

## Introduction

Prostate cancer has increased in incidence by 3% annually since 2014 (1). With significant advances in disease detection and treatment, the five-year survival rate of prostate cancer patients has now surpassed 97% (2). However, once patients develop resistance to standard androgen-deprivation therapy (ADT) and progress to castration-resistant prostate cancer (CRPC) or metastatic tumors, survival rates plummet to 33% (3). This stark contrast highlights the urgent need for new, more effective treatments for advanced prostate cancer patients. To address these challenges, innovative approaches for developing models that better recapitulate tumor progression and reveal the underlying mechanisms are required to evaluate and validate new therapeutic strategies.

In preclinical studies, LNCaP, PC3, and DU145 are three commonly used human metastatic prostate cancer cell lines, each with unique characteristics influencing their behavior and responsiveness to ADT. For example, LNCaP cells, derived from a supraclavicular lymph node metastasis site, express both androgen receptor (AR) and prostate-specific antigen (PSA), making them a valuable model for studying ADT-sensitive prostate cancer. In contrast, PC3 cells, originating from a vertebral metastasis, and DU145, derived from a brain metastatic site, lack expression of AR and PSA, rendering these cells resistant to ADT (4) and thus more aggressive. The CWR22Rv1 (22Rv1) cell line, another human prostate cancer cell line derived from xenografts of human metastatic prostate tumors, exhibits an intermediate profile with a mutated (overactive) AR and PSA expression (5), making it a unique tool for studying partial androgen signaling.

Although these human-derived cell lines provide insights into prostate cancer biology and response to therapy, their preclinical use is limited owing to immune rejection arising from species specificity. Therefore, various murine prostate cancers also have been isolated to enable a more comprehensive evaluation of the effects of therapeutics within the context of an intact immune system (6). For instance, commonly used murine cell lines include AR-expressing TRAMP-C1, TRAMP-C2, and TRAMP-C3, derived from 32-week prostatic adenocarcinomas of the probasin-SV40 T antigen-**Tr**ansgenic **A**denocarcinoma **M**ouse **P**rostate model (TRAMP) (7–9). In addition, the murine cell lines RM1, RM2, and RM9, developed by the Thompson group and deposited with the American Type Culture Collection (ATCC) in 1995, are AR-expressing, mesenchymal-like mouse prostate cancer cell lines. These cells were derived from 17-day-old mouse fetal urogenital sinuses and were retrovirally-transformed with the oncogenes *ras* and *myc* for studying androgen sensitivity (10, 11).

Both human and mouse prostate cancer cell lines, in addition to their key roles in advancing our understanding of tumor biology, typically also serve as platforms for testing novel therapeutic modalities, including oncolytic virotherapy. The concept of using viruses as a treatment for cancer was proposed over a hundred years ago, building on the first reported cancer cell remission in a patient with a natural viral infection (12, 13). Correspondingly, leukemia or lymphoma patients with a later viral infection also showed a period of tumor regression (14, 15). Thereafter, with the recent innovations made possible with genetic engineering, modified oncolytic viruses have been developed to reinforce their selective replication ability within cancer cells, particularly when equipped with a variety of therapeutic genes, as well as integrated strategies to disguise these viruses from the immune system (16, 17). For example, the oncolytic adenovirus H101, approved by Chinese regulatory agencies for patients with head and neck cancer as early as 2005, had deletions of the anti-apoptotic gene E1B and the immune evasion gene E3 (18). Similarly, T-VEC (herpes simplex virus 1; HSV-1), armed with human GM-CSF and deleted neurovirulence factors (ICP34.5 and ICP47), was the first documented modified oncolytic virus for glioma treatment and the first FDA-approved oncolytic therapy for unresectable stage III advanced melanoma patients in 2015 (19). At present, various viruses have been investigated in diverse cancer types, building on extensive studies that initially utilized cancer cell lines, demonstrating the potential of oncolytic virotherapy (including metastatic prostate cancer) for delivering different therapeutic genes and enabling combination therapies.

In this review, we explore the latest achievements in oncolytic virotherapy, a promising therapeutic approach that leverages viruses to selectively target and destroy cancer cells. We discuss commonly used oncolytic viruses in advanced prostate cancer from June 2019 to date, examining their mechanisms of action, therapeutic potential, and the challenges they face moving forward. Our focus is primarily on evaluation models for prostate cancer oncolytic virotherapy, with insights that may extend to other solid tumor types, aiming to showcase developments in the field that are likely to be adapted for prostate cancer.

## Key characteristics and mechanisms of oncolytic virus therapy

### Common types of viruses in prostate cancer treatment

**Figure 1:**
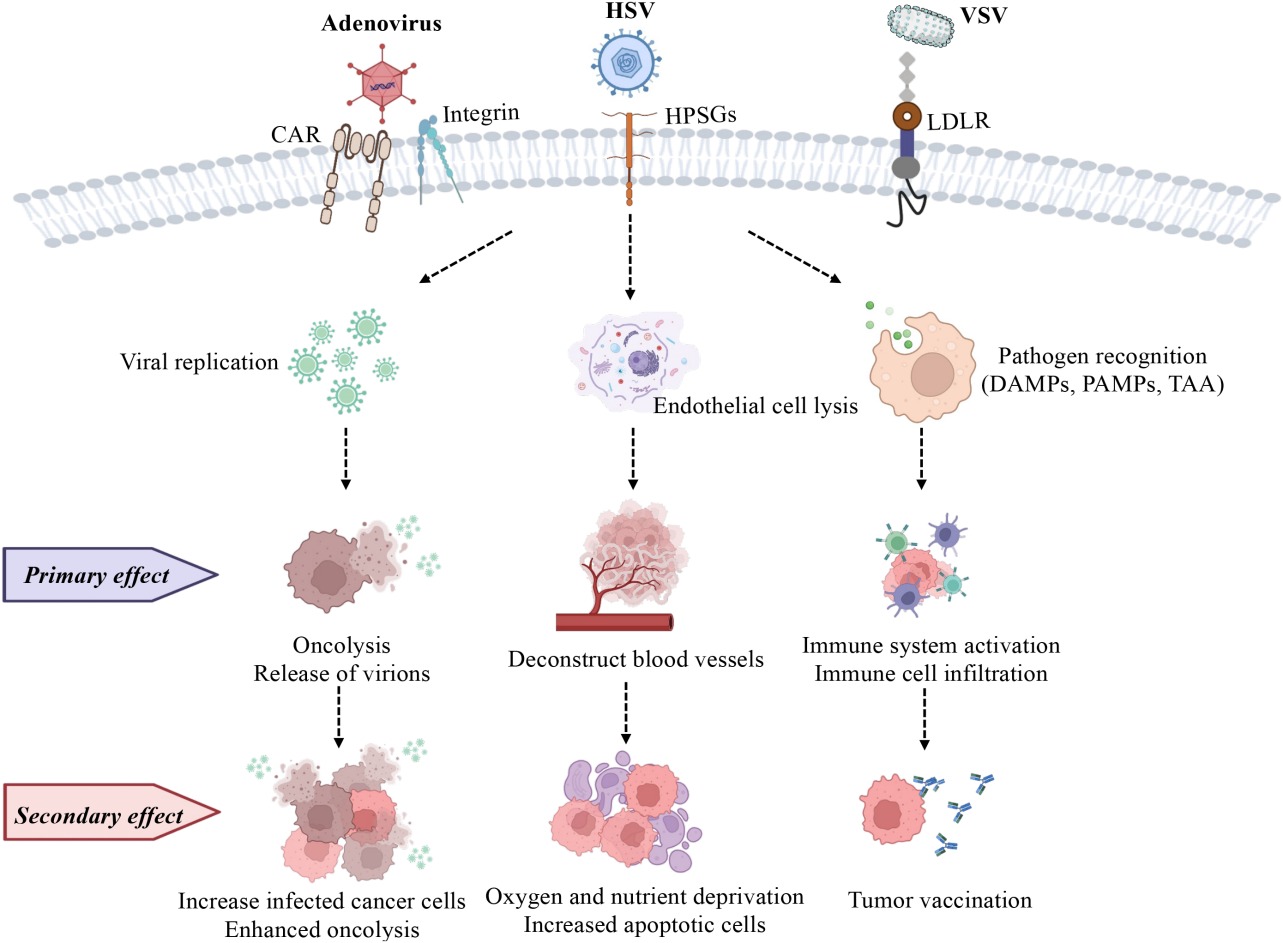
Commonly used oncolytic viruses (adenovirus, HSV, and VSV) are used to depict their effects on cancer cells. (1) Selective replication of OVs in cancer cells causes oncolysis (primary effect), and they further infect more cancer cells (secondary effect). (2) Endothelial cell death (anti-angiogenesis; primary effect) is a strategy for OVs to evade the immune system, meanwhile, the reduced vasculature decreases immune cell infiltration, and oxygen and nutrient supply (secondary effect), inhibiting tumor growth. (3) OVs alter the microenvironment by inducing both innate (primary effect) and adaptive immunity (secondary effect) as they respond to PAMPs, TAAs, and DAMPs. HPSGs, Heparan Sulfate Proteoglycans; LDLR, Low-Density Lipoprotein Receptor. Image created with BioRender.

Fourteen viruses, including RNA and DNA viruses, have been assessed in preclinical prostate cancer treatments over the past five years (**Table 1**). Among these, adenovirus, herpes simplex virus (HSV), and vesicular stomatitis virus (VSV) have been the most studied due to their genetic flexibility and high infectivity. While adenovirus, unlike HSV, lacks an envelope, both viruses consist of double-stranded DNA (dsDNA) and serve as mainstays in virotherapy because of their well-characterized genetic backgrounds and large capacity for carrying transgenes (52, 53). VSV is another frequently used oncolytic virus, composed of single-stranded RNA (ssRNA), for its rapid replication and abundant viral protein production that elicits robust immune responses (54). The main difference between DNA and RNA viruses is their mechanism of replication within host cells: DNA viruses require entry into the host cell nucleus, whereas positive-stranded RNA viruses can directly translate their proteins in the cytoplasm. In contrast, negative-stranded RNA viruses require an additional reverse transcription step in order to synthesize positive-stranded RNA before its proteins can be translated (55). Despite these differences, oncolytic viruses generally exert their cancer cell cytotoxicity through two main mechanisms: direct cell lysis (oncolysis), and indirect activation of anti-cancer immunity (immune-mediated) (**Figure 1**), with efficacy verified in both *in vitro* and *in vivo* studies, which have enabled these immune virotherapies to enter clinical trials (56).

**Table 1:** Organized oncolytic virus publication on prostate cancer within 5 years.

Models and Hosts Viruses	*in vitro* (2D culture)	*in vitro* (3D culture)	*in vivo*	Patients/samples	Host reservoir
**Measles**	PC3 (20)	–	–	–	Human
**Vaccinia Virus**	PC3 (21)	–	PC3 (21)	–	Human and mammals
**Alphavirus**	RM1 (22)	–	RM1 (22)	–	Human, Mammals, Marsupials, Birds, and Mosquitos
**Newcastle Disease Virus**	DU145/PC3 (23) RM9 (24)	- -	DU145 (23) RM9 (24)	- -	Birds (Avians), can infect Human
**Epizootic Hemorrhagic Disease Virus**	LNCaP/PC3 (25)	–	–	Patient samples (25)	Ruminants (Reoviridae)
**Orthoreovirus**	PC3 (26)		–	–	Vertebrates
**Zika Virus**	PC3 (27) PC3 (28)	- -	- -	- -	Monkey, Aedes mosquitos, and Human
**Parainfluenza Virus**	22Rv1 (29)	22Rv1 (29)	–	–	Human
**Chimpanzee Adenovirus 6**	RM1 (30)	–	RM1 (30)	–	Chimpanzee
**Sendai Virus**	–	–	–	Patients (31)	Mice, Rats, Hamsters, and Guinea pigs
**Reovirus**	TRAMP-C2/PC3/DU145 (32) 22Rv1/DU145/PC3 (33)	- MSK-PCa1/PDX from bone and liver metastasis (33)	TrampC2 (32) PC3 (33)	- Patient samples (33)	Vertebrates
**Herpes Simplex Virus (HSV)**	TRAMP-C2/DU145 (34) DU145 (35) PC3/LNCaP/22Rv1 (36)	Spheroid (34) - -	TrampC2/DU145 (34) - -	- - -	Human
**Vesicular Stomatitis Virus (VSV)**	LNCaP/PC3 (37) PC3 (38) DU145/LNCaP/PC3 (39) TRAMP-C2 (40)	- - -	LNCaP/PC3 (37) PC3 (38) - TrampC2 (40)	- - - Patient samples (40)	Indiana Vesicular Virus: Horse, Cattle, Pig, Sandflies, and Human
**Adenovirus**	DU145/PC3 (41) LNCaP/C4-2 (42) DU145 (43) DU145/LNCaP/PC3 (44) DU145/PC3 (45) PC3 (46) - DU145/LNCaP/PC3 (47) 22Rv1/PC3 (48) LNCaP/PC3 (49) PC3 (50)	TRAMP-C2 (42) - - - - - - - - - -	PC3 (41) 224B1/Ki-ras (42) DU145 (43) PC3 (44) PC3 (45) PC3 (46) - PC3 (47) PC3 (48) LNCaP (49) PC3 (50)	- - Patients (43) - - - Patients (51) - - - -	Human

### Cytotoxicity of oncolytic viruses

**Figure 2:**
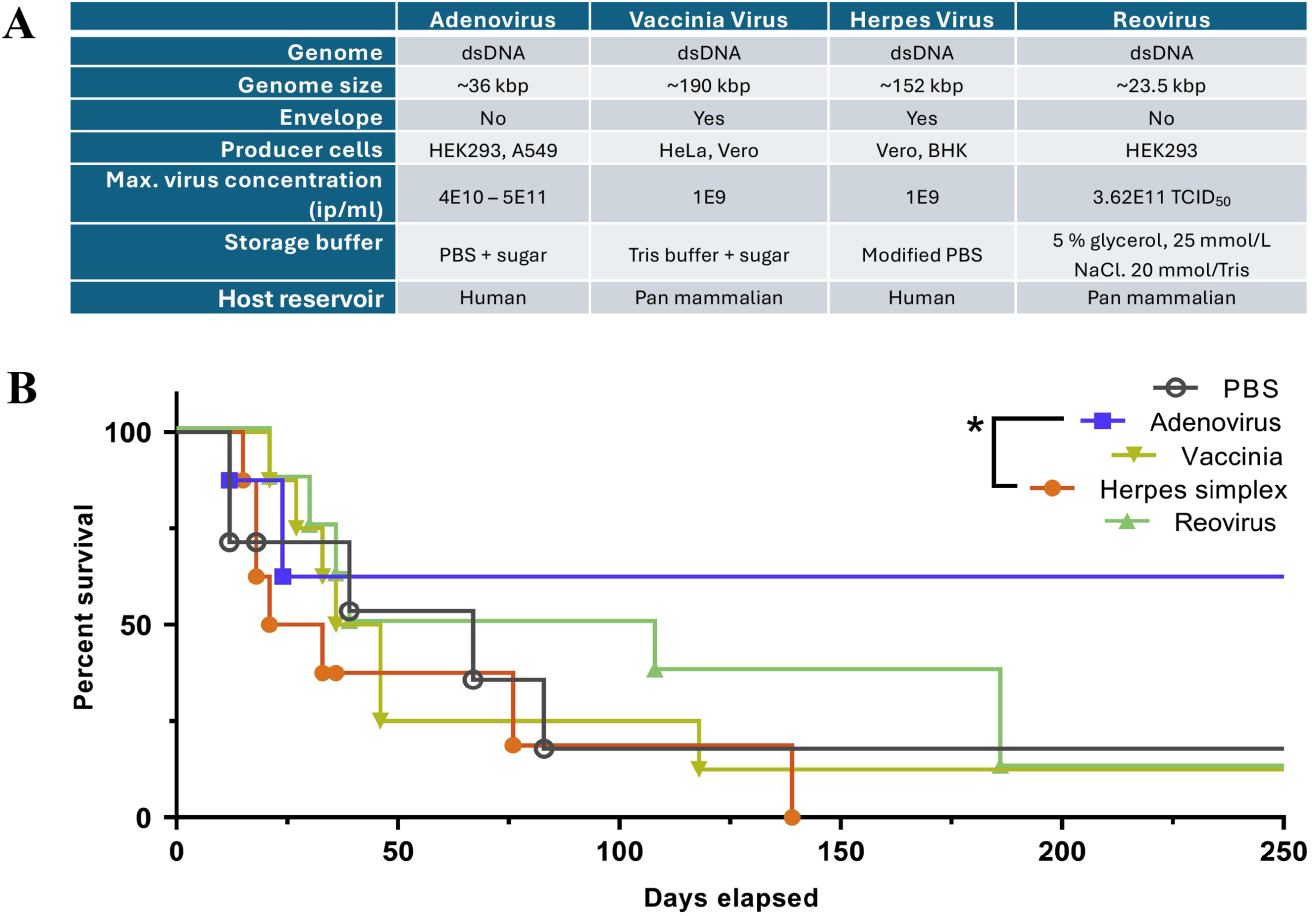
A compilation of oncolytic virus features for a brief comparison across OVs in **(A)**, modified from Ungerechts et al. (57), 2016 (https://pmc.ncbi.nlm.nih.gov/articles/PMC4822647/) and used under the Creative Commons Attribution (CC BY-NC-SA 4.0 International License). **(B)** A comparison of the survival of pancreatic adenocarcinoma-bearing hamsters treated with different OVs, including Adenovirus, Vaccinia Virus, Herpes Simplex Virus, and Reovirus148. The graph is derived from the original article by Cervera-Carrascon et al. (58).

The cytotoxicity of oncolytic viruses has been found to affect cancer cells through several mechanisms, including direct oncolysis, immune-mediated toxicity, and the disruption of tumor-associated blood vessels, all of which are processes that can assist in cancer cell elimination (**Figure 2**) (59). Upon viral infection, some key differences between healthy cells and tumor cells rather facilitate preferential virus replication. In normal cells, viral replication usually slows down metabolism and triggers the recruitment of leukocytes for viral clearance (60). Cancer cells, on the other hand, have developed ways to evade the immune system and avoid apoptosis, thereby supporting viral multiplication. These distinct cellular responses form the concept of oncolytic virotherapy, where viruses exhibit a natural tropism for replicating in cancer cells. Active multiplication drains the nutrients and energy of the host cells (61), causing direct oncolysis, a cell lysis process for virion release. Subsequently, tumor-associated antigens (TAAs), virus-associated antigens, and danger-associated molecular pattern molecules (DAMPs) are exposed, enhancing the recognition probability for phagocytosis by macrophages and dendritic cells (DCs), and facilitating antigen presentation to lymphocytes in lymph nodes. This process activates immune responses, resulting in immune-mediated indirect oncolysis (12). Additionally, to further promote replication and spread within tumor cells, some oncolytic viruses develop mechanisms to disrupt the host cells’ access to blood vessels, thus reducing nutrient supply and limiting the migration of immune cells to the tumor. Consequently, oncolytic viruses provide novel ways to robustly eliminate cancer cells, regardless of whether the cells were infected, by disrupting essential support systems such as the nutrient supply and migration routes (62, 63).

## Optimizing oncolytic viruses for prostate cancer treatment

### Viruses can exert cancer cell cytotoxicity as a monotherapy

**Figure 3:**
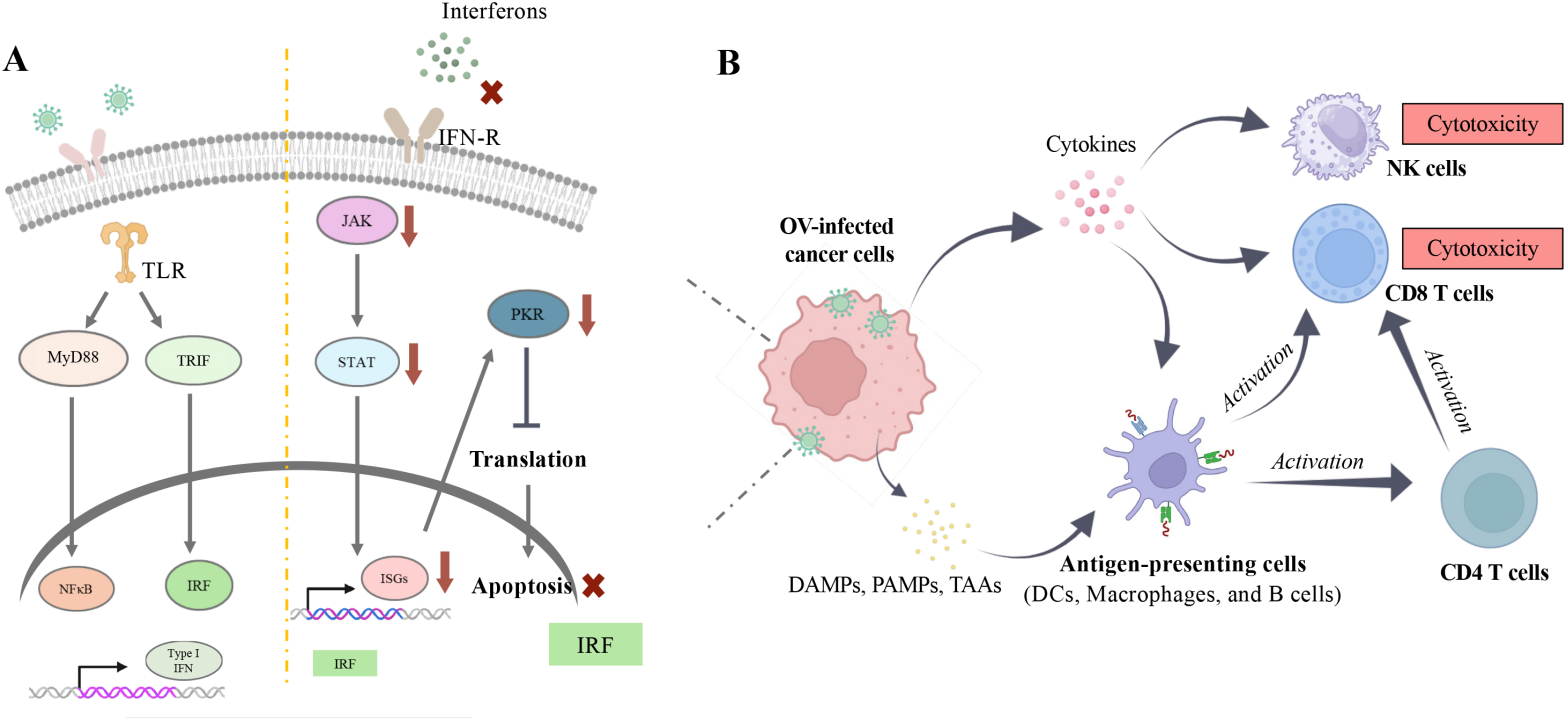
Immune modulation by oncolytic virus. **(A)** Upon viral infection, natural protective mechanisms in healthy cells can be activated once viral genetic material is recognized by Toll-like receptors (TLRs) on the endoplasmic reticulum (ER). This recognition triggers downstream signaling pathways, including MyD88/NFĸB and TRIF/IRF, which further lead to the active transcription of type I interferons (IFNs) (Left Panel). Under normal conditions, type I IFNs can initiate a cascade of immune responses through the JAK-STAT pathway to clear pathogens. However, in cancer cells, this response is impaired due to various mutations that allow them to evade detection by the immune system, such as becoming insensitive to IFN stimulation to avoid apoptosis (Right Panel). **(B)** Evasion of apoptosis facilitates viral replication within cancer cells and ultimately leads to oncolytic cell death. This process releases damage-associated molecular patterns (DAMPs), pathogen-associated molecular patterns (PAMPs), and tumor-associated antigens (TAAs), which enhance immune recognition. Images created with Biorender.

In most OV immunotherapies, cancer cell death is directly caused by the susceptibility of cancer cells to OV (lytic viral replication) and their failure to respond to anti-pathogen signals (17, 64). In healthy cells, various defense mechanisms are present to fight against pathogens, including viruses (65). For instance, one commonly observed pathogenic defense pathway is through type I interferon (IFN), secreted after pathogen detection (66). Upon type I IFN exposure, downstream signals are conveyed to the JAK-STAT or PKR pathways, inducing the transcription of effector genes such as interferon-stimulated genes (IRFs), including PKR, which can lead to apoptosis to limit infection (67, 68) (**Figure 3A**). However, cancer cells lose some of these abilities and evade immune surveillance. In a study published by Owen et al. in 2020, the loss of intrinsic IFN expression in tumor cells enables immune evasion and thus promotes prostate tumor progression to a more advanced state. By further restoring the presence of IFN through activation of HDAC, a greater number of tumor cells are eliminated by immune cells (69). Altogether, the primary attack of viruses, which results in the exposure of damage-associated molecule patterns (DAMPs), pathogen-associated molecule patterns (PAMPs), and tumor-associated antigens (TAAs) released from nonviable or apoptotic cells, drastically increases the number of targets/antigens for immune effectors (i.e. CD8+ T cells and antibodies) to recognize. Thus, this reveals another distinct trait of OV: alteration of the tumor microenvironment leading to elevated exposure of cancer cells to immunocytes (70–72) (**Figure 3B**).

Viruses can serve as an independent monotherapy, exerting cytotoxic effects on prostate cancer cells. For example, mammalian orthoreovirus (MRV), one of the most promising oncolytic viruses, has completed phase I to III clinical trials for various cancers (73, 74). In a 2021 study by Bussiere and Miller, MRV infection was shown to reduce HIF-1α levels during the early-stage viral infection in prostate cancer cells (26). As HIF-1α contributes substantially to cancer cell aggressiveness (75, 76), this finding uncovered a potential mechanism of cancer cell inhibition mediated by MRV infection. Another notable oncolytic virus, the M protein mutated vesicular stomatitis virus (M51R-VSV), has shown the ability to infect several human cancer cell lines and exert cytotoxic effects through elevated apoptosis, as reported in 2008 by Ahmed et al. (77). However, this effect was absent in one human prostate cancer cell line, PC3, due to the consistent expression of interferon-stimulated genes (ISG) (78). To overcome this limitation of PC3 cells, Bayne et al. demonstrated that by silencing MAP3K and CHD1, PC3 cells could become highly susceptible to M51R-VSV infection. Similarly, in a mouse model, significant tumor growth inhibition was observed in PC3 cells expressing short hairpin RNA against mitogen-activated protein kinase 3 and chromodomain helicase DNA binding protein (shMAP3K/CHD1) after M51R-VSV treatment (38). Therefore, although oncolytic viruses alone can induce cancer cell death, there are still limitations to their therapeutic potential. These most recent studies suggest that combination therapies may be crucial in augmenting their efficacy.

### Synergistic effects of modified oncolytic viruses and combination therapies

To enhance targeting specificity and therapeutic effects, the modification of oncolytic viruses with therapeutic genes or the use of combination therapies has been extensively studied with promising results. One example involves the oncolytic adenovirus DD3-ZD55-SPAG9, which uses a strategy of silencing sperm-associated antigen 9 (SPAG9), a protein involved in the MAPK signaling pathway and highly expressed in prostate cancer (79, 80), in the ZD55 backbone virus (an E1B55K-deleted adenovirus type serotype 5). Additionally, differential display code 3 (DD3) (81, 82), a promoter not expressed in normal prostate tissue, has been used to increase specificity in prostate cancer cell targeting (41). The results showed that DD3-ZD55-SPAG9 inhibited proliferation and migration in two human prostate cancer cell lines, PC3 and DU145. As combination therapies are widely used strategies in cancer therapeutics, DD3-ZD55-SPAG9 was combined with the chemotherapy drug docetaxel in both *in vitro* and *in vivo* systems, with results showing an even greater apoptotic effect on cancer cells. In this context, reovirus, another oncolytic virus demonstrating promising treatment outcomes, has also been investigated in combination with immune checkpoint blockade (PD-1) and immunomodulators (CD74). A study by Annels et al. demonstrated that unmodified reovirus, in combination with anti-PD-1 and anti-CD74 antibodies, significantly prolonged tumor growth inhibition (32). Immune profiling results showed an increase in chemokine receptors on the tumor cell surface, which could be recognized by T cells, NK cells, DCs, and B cells. This indicates that the reovirus infection altered the tumor microenvironment, facilitating greater immune cell infiltration. This change may further sensitize cancer cells to PD-1 and CD74 immuno-blockade therapies, leading to a significant reduction in tumor growth.

### Prolonging the sustainability of oncolytic viruses

Despite promising results, oncolytic viruses still face the challenge of maintaining their stability within biological systems (83). Immunity, as the body’s primary defense against foreign pathogens, creates a barrier to viral therapy regimens. Therefore, several novel carriers have been proposed to help protect and deliver the virus as part of combined therapeutic strategies. For example, mesenchymal stromal/stem cells (MSCs) have been reported to express limited MHC class I and lack expression of MHC class II, allowing them to evade immune surveillance during their transit in circulation (84, 85). This characteristic makes MSCs promising candidates for delivering viruses into the system, which enables more viral particles to reach the target site to exert their cytolytic effects. In a 2019 study by Muhammad et al., MSCs were used as carriers for oncolytic adenoviruses and demonstrated a significant reduction in tumor growth *in vivo*, along with increased apoptosis of cancer cells within tumor tissues (42). Besides MSCs, nanoparticles have emerged as a popular field of investigation in recent years, as they can enhance the bioavailability of drugs and facilitate targeted delivery to tumor sites (86). For example, Anjum et al. demonstrated that a nano-formulated measles virus, combined with the chemotherapy drug vincristine, induced cancer cell death and G2/M cell cycle arrest (20). The results showed that the encapsulated virus and vincristine were released in a sustained manner for over 72 hours, offering benefits such as potentially less frequent treatments for patients and prolonged cytotoxic effects against cancer cells.

## Platforms to determine oncolytic virus cytotoxicity in prostate cancer

### *In vitro* models

Two-dimensional (2D) cell culture is one of the most accessible and commonly used tools to verify the direct cytotoxicity of OV in cancer cells. Multiple cancer cell lines allow researchers to test various genetically modified viruses for specific cell-type targeting, further revealing mechanisms of OV action. For example, Catharino’s team reported metabolic changes in the PC3 human prostate cancer cell line following Zika virus infection, which induced cancer cell death and attenuated proliferative ability (27). Infected PC3 cells showed an increase in eicosatetraenoic acid (FA 20:5), an omega-3 polyunsaturated fatty acid, and its derivatives, oxylipins, which inhibited the phosphorylation of PYK2 and ERK, key proteins involved in cell signaling. This inhibition led to the accumulation of reactive oxygen species (ROS) after a 5-day infection, ultimately reducing the number of viable cancer cells. In another study, evidence suggested that the Newcastle virus (NDV), usually found in avians, was able to infect and replicate in multiple human cancer cells, including prostate cancer cells (23, 87, 88). Wang et al. revealed that NDV infection in human prostate cancer cell lines PC3 and DU145 led to the release of DAMPs, which promoted apoptosis and enhanced immunogenic cell death (ICD). Further, combining this infection with STAT3 inhibition increased the cancer cell-killing effects, with a notable increase in ICD markers, including calreticulin, HSP70/90, and HMGB1 (23). Considering the complexity of the *in vivo* environment, more advanced cell culture models, such as spheroids, organoids, and patient-derived tissues, can also be used to complement 2D studies and to better assess OV cytotoxicity.

Along these lines, a three-dimensional (3D) cell culture system provides a more complex tumor microenvironment that closely resembles the *in vivo* environment (89), offering a better model for assessing OV treatment efficacy. Spheroids, which are suspended clusters of cancer cells in layers, increase the difficulty of oncolytic viral infection, mimicking a more *in vivo*-like topology. P/V/F, a modified parainfluenza virus with a mutation in the P/V viral gene (encoding P: phosphoprotein and V: accessory protein) and an additional viral fusion protein (F), can selectively target prostate cancer cells (22Rv1) depending on the levels of type I interferon (IFN-I) present in culture. Kedarinath and Parks showed that as low as a multiplicity of infection (MOI) of 0.05 of P/V/F was able to infect 22Rv1 cells in 2D cell culture, and while viral infection efficiency decreased in the spheroid model, the modified virus still induced cancer cell death after an 18-hour infection (29). Additionally, two modified HSV strains, G47Δ and MG18L, have shown strong therapeutic effects on prostate cancer stem cell spheres. G47Δ includes deletions in the virulence gene γ34.5, a lacZ insertion to inactivate UL39, and deletion within the α47 gene to enable ICP47 production for T cell recruitment, while MG18L has deletion of US3 to activate NF-κB signaling and inactivation of UL39; both achieved IC50 values as low as MOI 0.09 and 0.021, respectively (34). Greater reductions in cancer cell growth were observed when G47Δ was combined with BKM120, a pan-class I PI3K inhibitor, in both *in vitro* and *in vivo* models, suggesting a synergistic therapeutic effect. While 3D cultures provide valuable structural complexity for further evaluating OV treatments, they cannot replicate the systemic interactions and immune dynamics of living organisms. As such, pre-clinical animal models are essential for ultimately advancing the therapeutic evolution of OV toward clinical relevance.

### Preclinical evaluation using animal models: insights and limitations

Pre-clinical animal models play a crucial role in providing a more comprehensive evaluation of therapeutics relative to the *in vitro* models discussed above. Among all species, rodent models are most frequently used in OV research, as they offer a broader perspective on treatment efficacy. This is particularly important since oncolytic virotherapies are highly dependent on stimulating the immune system to recruit effector cells.

Immune-competent mice can be especially useful, as they allow for detailed immune profiling and the assessment of synergistic effects with OVs, thereby shedding light on systemic responses. For example, Bai et al. demonstrated enhanced cytotoxicity using an oncolytic alphavirus combined with PD-L1-modulated Albendazole (ABZ) in immune-competent mice bearing RM-1 murine prostate tumors (22). Immune profiling and CD8+ T cell-deletion experiments showed that the increased cancer cell apoptosis was likely due to the greater CD8+ T cell infiltration detected in the tumor microenvironment. Additionally, the authors proposed that this combination therapy could sensitize tumors to immune checkpoint inhibitors such as anti-CTLA-4, thus promoting even more profound therapeutic outcomes. Similarly, McAusland et al. used RM-9 allograft C57BL/6 mice to evaluate the combination of oncolytic NDV and vanadyl sulfate, both of which possess anti-neoplastic properties, against melanoma (24). In these immune-competent mice, increased activation of NK cells and macrophages was detected. Interestingly, the combination treatment eliminated cancer cells through innate immunity, as no tumor-specific T cells were detected and the mice failed to respond when rechallenged with the same cancer cells. These mouse models are thus very valuable because they possess intact immune systems, enabling a more inclusive assessment of OV-based therapies. However, there are still notable genetic differences between mouse and human prostate cancer cells, which limit one’s ability to fully recapitulate human disease. Additionally, mice might not be permissive hosts for some oncolytic viruses, potentially leading to an underestimation of their therapeutic effects. To address these limitations, immune-compromised mice are frequently used in OV studies involving human cancer cell line implantation (90).

Immune-compromised mice are commonly used in studies that aim to mimic the human tumor microenvironment through xenografting of human prostate cancer cells. NCG mice, which lack T cells, B cells, and NK cells, are frequently used in OV studies as they greatly minimize graft-versus-host rejection. These mice are named for their genetic modifications: **N**OD (non-obese diabetic), **C**B17 background, and targeted deletion of the **G**amma chain (common cytokine receptor γ-chain). In a study by Fang et al. (2023), NCG mice were implanted subcutaneously (s.c.) with DU145 human prostate cancer cells and treated with a combination therapy of Ad5Ki67-C3, an adenovirus serotype 5 (Ad5) driven by the Ki67 promoter to express CCL5, interleukin 12, and interferon-γ (43). This treatment, combined with radiation, synergized towards a prolonged survival rate and significant tumor size reduction over 60 days. Moreover, long-term immunity against the same cancer cells was observed when the mice were re-challenged. Nu/nu mice (nude mice or athymic mice), while deficient in T cells due to a homozygous mutation in *Foxn1* causing thymic underdevelopment, are also frequently used in OV studies. One example is a study examining the efficacy of RCAd11pADP, an oncolytic adenovirus serotype 11b vector equipped with an adenovirus death protein (ADP). This virus demonstrated cytotoxic effects in both cell culture and PC3 xenograft mouse models (44). In BALB/c nude mice, increased apoptotic cancer cells were observed four weeks after two virus injections. In addition, elevated mRNA expression of viral E1A and hexon proteins indicated active viral replication within the tumor and its potential to suppress tumor growth. Similarly, Mao et al. (45) validated the apoptotic potential of ZD55-IL-24, an oncolytic adenovirus expressing the antitumor gene mda-7/interleukin-24, through both *in vitro* and *in vivo* studies. Elevated expression of Caspase-3 and Caspase-8 was detected 18 days post-ZD55-IL-24 treatment in prostate cancer cells and in PC3 tumor-bearing BALB/c nude mice, specially when combined with radiation. Although some immune-compromised mice retain partial immune function, such as innate immunity and innate-like T cells, the majority are athymic and lack diverse T cell populations (91). Given that T cells are crucial for the cytotoxic elimination of cancer cells, particularly in oncolytic virotherapy, results from these models might not fully capture the therapeutic potential observed in immune-competent systems.

With the continuous advancement of mouse models, humanized mice have emerged as some of the most relevant systems for mimicking key aspects of human biology. Humanized mice can be established using immune-deficient NSG mice transplanted with human hematopoietic stem cells (HSCs), human fetal thymic and liver tissue (BLT model), or through the injection of human peripheral blood mononuclear cells (PBMCs). These approaches temporarily allow researchers to analyze disease models and obtain human-relevant immunological responses (92). For example, Zafar et al. (2021) (46), used PBMC-humanized mice to evaluate a novel oncolytic adenovirus (Ad3-hTERT-CMV-hCD40L), equipped with human CD40L to stimulate dendritic cells (DCs). Mice treated with the hCD40L-expressing adenovirus showed significant tumor reduction and prolonged survival, with even greater effects observed in the presence of DCs. However, a key limitation of humanized mice is that they typically develop graft-versus-host disease (GvHD) around 40 days post-transplantation (93). This short experimental window poses challenges, especially in distinguishing immune responses induced by OV therapies from those caused by immune rejection.

In rodent systems, OV experiments often require multiple models to piece together a complete picture of how the virus interacts with both the tumor and the whole organism. This is largely due to species specific differences in viral susceptibility and compatibility with implanted cancer cells. While multiple mouse models may be needed to assess different aspects of OV function, a single hamster model can sometimes provide more comprehensive insights, especially for OVs that do not replicate efficiently in rodents, such as *adenovirus* sp*ecies C (* 94). For instance, Li et al. observed glioma growth inhibition in hamsters following infection with Ad-TD-nsIL-12 (an Ad5 with three deleted genes and producing non-secreting interleukin 12). Viral E1A expression was detectable 12 days post-infection, indicating successful viral propagation in the hamster model, consistent with *in vitro* results (95). However, in the context of prostate cancer research, some limitations arise. For example, in a study by Koodie et al. (2019), a modified adenovirus with a chimeric fiber (Ad5/3) was used to improve viral entry into advanced prostate cancer cells, which often exhibit reduced expression of the coxsackie-adenovirus receptor (CAR), the primary receptor for adenovirus subgroup C (96). The results showed a decrease in Ad5/3 permissibility in hamsters (97), suggesting that this model may not be ideal for evaluating such vectors. Therefore, while the hamster model provides an immune-competent environment and supports viral propagation, making it advantageous over some mouse models, it might not fully apply to human prostate cancer studies. These limitations stem from phenotypic differences between human and hamster prostate cancer cell lines (98), as well as a lack of established hamster allograft disease models. To gain further insight into immune responses following OV infection, patient-derived samples might offer the most accurate and clinically relevant representation prior to human trials.

### *Ex vivo* models using patient-derived tissues for OV research

Currently, no single model can fully recapitulate the complexity of prostate cancer tumors in patients, as preclinical systems have limited capacity to include all components of the tumor microenvironment. To overcome this limitation, patient-derived tumor tissues have become a valuable tool for more closely mirroring the biological characteristics of human cancers prior to clinical trials (99). In the same study mentioned earlier involving humanized mice, the authors further investigated the mechanisms underlying tumor reduction following synergistic OV and DC treatment using patient-derived prostate cancer tissues. Three days after viral infection, these tissue samples showed a notable elevation in DC maturation markers, including CD80, CD83, and CD86, along with a higher number of mature DCs in culture. In addition, significant upregulation of pro-inflammatory markers, such as IL-2, IL-12, TNF-α, and granzyme B, was detected in the culture media (46). A 2021 study by van de Merbel et al. further demonstrated the utility of patient-derived tissue slices and a xenograft mouse model to evaluate the therapeutic potential of jin-3, a reovirus variant with a spontaneous mutation in the Sigma-1 spike protein, allowing JAM-A (junction adhesion molecule A) independent infection of tumor cells (33). In tissue slices, time-course analysis confirmed viral replication, while in the patient-derived xenograft (PDX) mouse model, jin-3 treatment led to tumor shrinkage and widespread detection of the viral protein Sigma-3. Moreover, increases in apoptotic cell counts and decreases in proliferative markers were reported, supporting the virus’s cytotoxic activity. While human-derived samples might be the ideal testing platform for evaluating OV therapies before clinical trials, one key limitation remains, which is the scarcity and limited availability of these samples (100).

### Focus on five-year clinical trials of OVs in prostate cancer

Various viruses have been designed and evaluated for prostate cancer treatment; however, due to the gaps in human-relevant research models mentioned above, few have progressed to clinical trials. Based on the latest updates from *ClinicalTrials.gov*, there have been only nine OV clinical trials in prostate cancer worldwide within the last five years. Four types of viruses have been included in these trials: coxsackievirus, vaccinia virus, reovirus, and adenovirus, which together comprise the majority of viruses utilized (**Table 2**). While not every clinical trial result is publicly available, only those with accessible data are discussed here, including clinical trials publications of oncolytic coxsackie virus, oncolytic adenovirus, and oncolytic vaccinia virus.

**Table 2:** 5-year clinical trials on prostate cancer.

Virus	Clinical trial number	Phase	Modification	Prostate cancer stages	Status	Location
***ETBX-071 (*** 101) (Adenovirus)	NCT06765954	Phase II	• Deleted E1, E2b, and E3 region • Encoded prostate-specific antigen (PSA)	Nonsurgical high-risk prostate cancer patients	Not yet recruiting	Not provided
***ORCA-010 (*** 102) (Adenovirus)	NCT04097002	Phase I/IIa	• E1AΔ24 deletion • Infectivity-enhancing fiber RGD modification	Treatment-naïve patients with localized tumor	Active; not recruiting	Canada/Netherlands
***ONCOS-102 (*** 103) (Adenovirus)	NCT03514836	Phase I/II	• Immunostimulatory cytokine - GM-CSF	Castration-resistant advanced metastatic prostate cancer	Terminated; insufficient accrual	Finland/Czechia
***Ad5-yCD/mutTKSR39rep-hIL12 (*** 104) (Adenovirus)	NCT02555397	Phase I	• Human interleukin-12 • Two suicide fusion genes: Yeast cytosine deaminase (yCD) and a mutant form of herpes simplex virus type 1 thymidine kinase (HSV-1 TKSR39)	Locally recurrent prostate cancer after definitive radiotherapy	Completed (results)	United States
***AdNRGM (*** 105) (Adenovirus)	NCT04374240	Phase I	• E1-E3 deleted, replication deficient • Human GMCSF gene	Local recurrence of prostate cancer following radical radiotherapy	Completed	United Kingdom
***Ad5-SGE-REIC/Dkk-3 (*** 106) (Adenovirus)	NCT01931046	Phase I/IIa	• Super gene expression (SGE) system • Tumor suppressor gene dickkopf-3	Localized prostate cancer	Completed	United States
***Reolysin (*** 107) (Reovirus)	NCT01619813	Phase II	• N/A	Metastatic or locally recurrent prostate cancer	Completed	Canada
***MVA-brachyury-TRICOM (*** 108) (Vaccinia virus)	NCT02179515	Phase I	• Replication-deficient, attenuated • Brachyury • Triad of human costimulatory molecules (B7.1, LFA-3, and ICAM-1)	Metastatic or unresectable locally advanced malignant solid tumor	Completed (results)	United States
***CVA21 (*** 109) (Coxsackie virus)	NCT00636558	Phase I	• N/A	Stage IV solid tumor	Completed	Australia

The phase I clinical trial of unmodified oncolytic coxsackie virus, CVA21 (V937), completed in 2019, evaluated its effect on various solid tumors, including prostate cancer (110). Four patients with metastatic castration-resistant prostate cancer received CVA21 on days 1, 3, and 5 during the first 21-day cycle, followed by a single dose on day 1 of each of the next eight cycles. No dose-limiting toxicities (DLTs) were reported during monotherapy, and although no specific patient outcomes were indicated, an increase in V937 antibodies in serum was observed during the treatment cycles. This phase I clinical trial established the safety profile of CVA21 across several cancer types and provided data on the tolerance of repeated intravenous injections. However, as it was a pilot study assessing dosage tolerance, a larger cohort of prostate cancer patients would be needed to solidify the results and fully evaluate its therapeutic potential (109).

The modified oncolytic adenovirus Ad5-yCD/*mut*TK_SR39_ *rep*-hIL-12 was evaluated for its dosage tolerance and safety in a phase I clinical trial involving 15 patients with localized recurrent prostate cancer. A single intraprostatic dose of the virus, ranging from 1 × 10^10^ to 1 × 10^12^ viral particles, was administered on the first day of the trial, then followed by seven days of 5-fluorocytosine (5-FC) and valganciclovir (vGCV) chemotherapy (51). The Ad was designed to express cytosine deaminase (CD) and HSV thymidine kinase (TK), which convert the pro-drugs 5-FC and GCV into toxic agents that eliminate the cancer cells by interfering with DNA synthesis. The safety of this treatment was confirmed, with no reported DLTs, and 92% of side effects were classified as either grade 1 (mild) or grade 2 (moderate). Also, elevated levels of CD3^-^CD56^+^ NK cells, CD3^+^CD4^+^ T helper cells, and CD3^+^CD8^+^ cytotoxic T cells were observed in patients, suggesting immune modulation in peripheral blood due to IL-12 expression from the Ad (104).

The modified oncolytic vaccinia Ankara (MVA) virus encoding TAA (brachyury) and a triad of T-cell co-stimulatory molecules (TRICOM), MVA-Brachyury-TRICOM, also completed a phase I clinical trial involving various cancers, including prostate cancer, to evaluate the safety of three different virus dosages (111). Preliminary results indicated changes in immune cell profiles, especially an increase in CD8^+^ T cells. Nonetheless, serious side effects were observed in patients receiving higher dosages. While only three prostate cancer patients were enrolled out of 38 total participants, no specific outcome data for these patients has been reported yet (108). Further studies focusing specifically on prostate cancer could benefit from larger patient enrollment and more detailed analyses, particularly regarding prognosis and response to treatment.

## Trends of oncolytic viral therapy in cancer

Recent studies highlight the promising future of oncolytic viruses, with significant research focusing on adenovirus, herpes simplex virus, vaccinia virus, vesicular stomatitis virus, and reovirus. Here, a brief discussion covers the distinct traits of these oncolytic viruses and addresses several challenges that require attention: short therapeutic windows *in vivo*, insufficient accumulation in tumors, restricted delivery routes, and the need to better characterize their overall safety profiles. Regardless, advancements in oncolytic viral therapy research hold great promise for a wide variety of cancer types. By leveraging the unique properties of these viruses, the field can develop more effective immunotherapies, leading to improved treatment outcomes for patients, especially those whose tumors are resistant to immune checkpoint inhibitors.

### Why adenovirus, herpes simplex virus, and more

Our literature review reveals that certain viruses are used more frequently than others due to their natural characteristics. For instance, reovirus, a dsRNA virus, naturally infects transformed cells, which synthesize viral proteins more efficiently (112, 113). Similarly, VSV, a negative-sense RNA virus, preferentially infects cancer cells with defective IFN pathways, which reduce their antiviral response and make them more permissive to infection (114). HSV also has a preference for cancer cells with mutated Ras pathways (115) and has the added advantage of a large genome capacity for further modification (19, 116). Likewise, adenovirus (117, 118) and vaccinia virus (VV) (119, 120) have relatively large genome capacities, allowing modifications to enhance their cytotoxicity against tumor cells.

However, therapeutic evaluation models can be challenging for these viruses. As previously mentioned, human adenovirus predominantly infects humans, making *in vitro* tests straightforward. However, when it comes to pre-clinical animal studies, limitations arise: there is no single model that can offer comprehensive validation of these treatments. Rodents, even across diverse strains, are generally non-permissive to infection or replication of these viruses, making it difficult to accurately assess therapeutic efficacy and safety *in vivo*. It has been reported that IFNs induced mouse Mx1 expression, which suppresses HSV replication in mice (121). In this context, immunity can act as a double-edged sword in oncolytic virotherapy. While a full immune response, including antiviral mechanisms, can recapitulate the human immune system, the inhibition of HSV replication also can reduce therapeutic effects. Therefore, careful consideration is needed when examining and choosing different models (122).

### Neutralization and clearance of OV within the system

Throughout history, humans have co-evolved with viruses, and it may come as a surprise that over 50% of the human genome originates from viruses and transposable elements. These genetic materials, acquired through horizontal gene transfer, crossover and recombination, and transformation, have significantly shaped who we are today (123), including our immunity. Over time, the diversity of the major histocompatibility complex (MHC) has further impacted T cell and B cell specificity, as well as antibody production, thus strengthening our immune response to pathogens (124, 125). In a study by Alemany et al. in 2000 (10), it was reported that 10^10^ transducing units (t.u.) of adenovirus serotype 5 particles have a half-life of less than 2 minutes following vena cava injection, with viral sequences being cleared by Kupffer cells in the liver within 24 hours (126). To prolong circulation time, scientists have genetically engineered viruses, equipping them with inhibitors of CD8+ T cells (127) or NK cell activation (128). Similarly, disguising the virus using polyethylene glycol (PEG) (for Ad and VSV) or using mesenchymal stromal/stem cells (MSCs) as viral carriers has been shown also to prevent rapid clearance, thereby extending circulation time (126, 129–131). Protected virus not only extend their effective duration in the system but also enhance their migration toward target sites by reducing accumulation in the liver (129, 131). While rapid neutralization of oncolytic viruses might seem counter-intuitive as a therapeutic strategy, it could act to enhance immune cell infiltration into tumors, thereby improving therapeutic outcomes (132, 133).

To avoid virus loss during migration and inefficient OV accumulation at target sites (134), studies have focused on enhancing targeting toward cancer cells. For example, tumor-specific proteins have been expressed on the surface of tumor cells, and “tagging” the tumor surface or other aspects of its unique microenvironment can help direct the OV to target sites (135, 136). However, the majority of OVs are still lost during routing towards tumors, leading to an underestimated treatment effectiveness and a shift from clinically favorable intravenous injection to the more locally-limited intratumorally injection (24, 137–140). As a result, various delivery tools, including nanoparticles, vesicles, and cells (141), are being explored by several groups to attempt to overcome these challenges.

### Safety of OV therapy and authorized OV therapy status

Another way to address delivery challenges is through administering a concentrated dosage of the virus. However, this approach could trigger an acute immune response (cytokine storm), resulting in immune reactivity, organ damage (142), and increased risk of infection depending on the type of virus (143). Hence, combination therapy provides a promising solution to enhance treatment efficacy (144) without increasing the virus dose injected. Encouraging results from preclinical studies using combination therapies have already positively influenced the number of clinical trials. For example, as of January 2025, there were 52 actively recruiting OV-related clinical trials across various phases worldwide. These trials include engineered OVs as monotherapies and as adjuvants in combination therapies for different types of cancer. Moreover, several oncolytic viruses have been approved by regulatory authorities, including T-VEC (HSV1 armed with human GM-CSF and deleted neurovirulence factors ICP34.5 and ICP47; Australia, Europe, Israel, and the USA), ECHO-7 (echovirus; Armenia, Georgia, and Latvia), Teserpaturev (*Δ47*, *γ34.5*, and *ICP6* triple gene-mutated HSV1; Japan), and H101 (*E1B-* and *E3-* deleted adenovirus serotype 5; China) (145). However, due to partially unelucidated mechanisms of action against tumors, pharmacovigilance continues to be closely monitored for these clinically approved regimens. According to the latest publication utilizing the U.S. FDA Adverse Event Reporting System (FAERS) database to retrospectively analyze 1138 patients receiving T-VEC, the most commonly seen side effects matched prescribing information or previously reported cases. However, four unexpected adverse events (sepsis, encephalitis, syncope, and lymphadenopathy) were identified. Additionally, 10% of patients receiving T-VEC discontinued treatment, and 2% had life-threatening conditions (146), emphasizing the need for further comprehensive clinical studies to minimize potential harm to patients.

### What types of cancer can benefit most from OV therapy?

The concept of “hot” and “cold” tumors was first introduced in 2006, whereby tumors can be classified based on the distribution of immune cells in the tumor microenvironment (147). With immunoscoring of tumor-infiltrating lymphocytes (TILs), “hot” tumors have been defined by the presence of TILs and a high incidence of tumor mutations, while “cold” tumors have been considered to be the opposite (148). Cold tumors develop mechanisms to evade immune surveillance, including the expression of PD-L1 to inhibit CD8^+^ T cell activation, expression of CD47 to escape from dendritic cell recognition (149), and decreased expression of MHC-I (150). While CAR-T (chimeric antigen receptor T cell) therapy, targeting CD19 and expressing co-stimulatory 4-1BB with CD3ζ signaling domain (151), has achieved great success in enhancing immune responses and tumor regression in hematologic cancers (lymphomas and leukemia), challenges remain in treating CD19-negative tumors, T cell malignancies, and solid tumors (152). Thus, various inflammatory cytokine-expressing CAR T cells have been studied in recent years to target solid tumors, with positive preclinical results (153). Yet, the immunosuppressive tumor microenvironment of cold tumors and the lack of distinct tumor-specific targets have remained as significant barriers (152).

Oncolytic viruses, with their unique ability to remodel the immunosuppressive tumor microenvironment (154, 155), have become promising candidates for treating challenging tumor types. Examples include increased IFNγ infiltration observed in head and neck squamous cell carcinoma (HNSCC) preclinical models treated with a TGFβ inhibitor-expressing VV, making the tumor more responsive to treatment (156). The oncolytic adenovirus Delta-24-RGDOX, also induced increased IDO in human and murine glioma cells (157). Additionally, Hirigoyen et al. reported enhanced extracellular vesicle (EV) secretion by VSV-infected human melanoma cells, which amplified CD8 T cell cytotoxicity when incubated with EVs (158). Thus, the potential and plasticity of oncolytic viruses are under investigation in various cancers, particularly in difficult-to-treat cancers where delivery of therapeutic agents is hindered by the blood-brain barrier (BBB), such as immune-cold glioblastomas (159). In these contexts, OVs have been considered revolutionary.

While some cancer types are identified as immune ‘hot’ or highly responsive to immune checkpoint inhibitors (ICIs), such as melanoma, it remains difficult to predict patient prognosis, as only about one-third of patients respond to ICIs (160). Vareki et al. have unveiled that tumor mutation profiles can further explain clinical responses, as tumors with high mutational loads may express more tumor-associated antigens, enhancing immunocyte recognition and recruiting more immune effectors (161). In contrast, immune “cold” malignancies, such as prostate cancer, pancreatic cancer, and neuroblastoma, typically have low mutational loads and poor responses to immunotherapy, prompting ongoing research seeking breakthroughs. The use of OVs to transform the tumor microenvironment (162–164) can enable better control of these cancers, with promising results reported from both pre-clinical (165, 166) and clinical studies (167, 168).

In summary, OVs excel as therapeutics across multiple dimensions, including selective targeting and replication in cancer cells, primary oncolytic activity, secondary immune-mediated lysis, and a high genomic capacity for delivering multiple therapeutic genes. Promising outcomes have been demonstrated *in vitro*, *in vivo*, *ex vivo*, and in clinical studies. However, several limitations remain to be addressed, including marginal virus accumulation at tumor sites (169), restricted administration routes, and incompletely elucidated cytotoxic mechanisms. We envision that continued research into optimizing delivery strategies, enhancing tumor specific accumulation, and clarifying mechanisms of action will be critical to fully harness the therapeutic potential of OVs in prostate cancer clinical oncology.
