# Correction: Experimental models for developing oncolytic virotherapy for metastatic prostate cancer

**DOI:** 10.3389/fimmu.2025.1665709

**Published:** 2025-08-11

**Authors:** Ying-Cheng Chen, Marxa Leão Figueiredo

**Affiliations:** Department of Basic Medical Sciences, College of Veterinary Medicine, Purdue University, West Lafayette, IN, United States

**Keywords:** oncolytic virus, metastatic prostate cancer, immunotherapy, virotherapy, experimental models

There was a mistake in **Table 1** as published. There should be a separation slash between “Models and Hosts” and “Viruses”. The corrected **Table 1** appears below.

**Table 1:** Organized oncolytic virus publication on prostate cancer within 5 years.

Models and Hosts Viruses	*in vitro* (2D culture)	*in vitro* (3D culture)	*in vivo*	Patients/samples	Host reservoir
**Measles**	PC3 (20)	–	–	–	Human
**Vaccinia Virus**	PC3 (21)	–	PC3 (21)	–	Human and mammals
**Alphavirus**	RM1 (22)	–	RM1 (22)	–	Human, Mammals, Marsupials, Birds, and Mosquitos
**Newcastle Disease Virus**	DU145/PC3 (23) RM9 (24)	- -	DU145 (23) RM9 (24)	- -	Birds (Avians), can infect Human
**Epizootic Hemorrhagic Disease Virus**	LNCaP/PC3 (25)	–	–	Patient samples (25)	Ruminants (Reoviridae)
**Orthoreovirus**	PC3 (26)		–	–	Vertebrates
**Zika Virus**	PC3 (27) PC3 (28)	- -	- -	- -	Monkey, Aedes mosquitos, and Human
**Parainfluenza Virus**	22Rv1 (29)	22Rv1 (29)	–	–	Human
**Chimpanzee Adenovirus 6**	RM1 (30)	–	RM1 (30)	–	Chimpanzee
**Sendai Virus**	–	–	–	Patients (31)	Mice, Rats, Hamsters, and Guinea pigs
**Reovirus**	TRAMP-C2/PC3/DU145 (32) 22Rv1/DU145/PC3 (33)	- MSK-PCa1/PDX from bone and liver metastasis (33)	TrampC2 (32) PC3 (33)	- Patient samples (33)	Vertebrates
**Herpes Simplex Virus (HSV)**	TRAMP-C2/DU145 (34) DU145 (35) PC3/LNCaP/22Rv1 (36)	Spheroid (34) - -	TrampC2/DU145 (34) - -	- - -	Human
**Vesicular Stomatitis Virus (VSV)**	LNCaP/PC3 (37) PC3 (38) DU145/LNCaP/PC3 (39) TRAMP-C2 (40)	- - -	LNCaP/PC3 (37) PC3 (38) - TrampC2 (40)	- - - Patient samples (40)	Indiana Vesicular Virus: Horse, Cattle, Pig, Sandflies, and Human
**Adenovirus**	DU145/PC3 (41) LNCaP/C4-2 (42) DU145 (43) DU145/LNCaP/PC3 (44) DU145/PC3 (45) PC3 (46) - DU145/LNCaP/PC3 (47) 22Rv1/PC3 (48) LNCaP/PC3 (49) PC3 (50)	TRAMP-C2(42) - - - - - - - - - -	PC3 (41) 224B1/Ki-ras (42) DU145 (43) PC3 (44) PC3 (45) PC3 (46) - PC3 (47) PC3 (48) LNCaP (49) PC3 (50)	- - Patients (43) - - - Patients (51) - - - -	Human

The virus particle unit number was incorrect.

A correction has been made to the section **Platforms to determine oncolytic virus cytotoxicity in prostate cancer**, *Focus on five-year clinical trials of OVs in prostate cancer, paragraph 3:*

“The modified oncolytic adenovirus Ad5-yCD/*mut*TK_SR39_ *rep*-hIL-12 was evaluated for its dosage tolerance and safety in a phase I clinical trial involving 15 patients with localized recurrent prostate cancer. A single intraprostatic dose of the virus, ranging from 1 × 10^10^ to 1 × 10^12^ viral particles, was administered on the first day of the trial, then followed by seven days of 5-fluorocytosine (5-FC) and valganciclovir (vGCV) chemotherapy (51). The Ad was designed to express cytosine deaminase (CD) and HSV thymidine kinase (TK), which convert the pro-drugs 5-FC and GCV into toxic agents that eliminate the cancer cells by interfering with DNA synthesis. The safety of this treatment was confirmed, with no reported DLTs, and 92% of side effects were classified as either grade 1 (mild) or grade 2 (moderate). Also, elevated levels of CD3^-^CD56^+^ NK cells, CD3^+^CD4^+^ T helper cells, and CD3^+^CD8^+^ cytotoxic T cells were observed in patients, suggesting immune modulation in peripheral blood due to IL-12 expression from the Ad (104).”

The virus particle unit number was missing.

A correction has been made to the section **Trends of oncolytic viral therapy in cancer**, *Neutralization and clearance of OV within the system, paragraph 1:*

“Throughout history, humans have co-evolved with viruses, and it may come as a surprise that over 50% of the human genome originates from viruses and transposable elements. These genetic materials, acquired through horizontal gene transfer, crossover and recombination, and transformation, have significantly shaped who we are today (123), including our immunity. Over time, the diversity of the major histocompatibility complex (MHC) has further impacted T cell and B cell specificity, as well as antibody production, thus strengthening our immune response to pathogens (124, 125). In a study by Alemany et al. in 2000 (10), it was reported that 10^10^ transducing units (t.u.) of adenovirus serotype 5 particles have a half-life of less than 2 minutes following vena cava injection, with viral sequences being cleared by Kupffer cells in the liver within 24 hours (126). To prolong circulation time, scientists have genetically engineered viruses, equipping them with inhibitors of CD8+ T cells (127) or NK cell activation (128). Similarly, disguising the virus using polyethylene glycol (PEG) (for Ad and VSV) or using mesenchymal stromal/stem cells (MSCs) as viral carriers has been shown also to prevent rapid clearance, thereby extending circulation time (126, 129–131). Protected virus not only extend their effective duration in the system but also enhance their migration toward target sites by reducing accumulation in the liver (129, 131). While rapid neutralization of oncolytic viruses might seem counter-intuitive as a therapeutic strategy, it could act to enhance immune cell infiltration into tumors, thereby improving therapeutic outcomes (132, 133).”

The original version of this article has been updated.

